# Recombinant Enzymatic Redox Systems for Preparation of Aroma Compounds by Biotransformation

**DOI:** 10.3389/fmicb.2021.684640

**Published:** 2021-06-24

**Authors:** Viktor Varga, Vladimír Štefuca, Lenka Mihálová, Zdenko Levarski, Eva Struhárňanská, Jaroslav Blaško, Robert Kubinec, Pavel Farkaš, Vladimír Sitkey, Ján Turňa, Michal Rosenberg, Stanislav Stuchlík

**Affiliations:** ^1^Department of Molecular Biology, Faculty of Natural Sciences, Comenius University, Bratislava, Slovakia; ^2^Institute of Biotechnology, Faculty of Food and Chemical Technology, Slovak University of Technology, Bratislava, Slovakia; ^3^Science Park of Comenius University, Bratislava, Slovakia; ^4^Department of Analytical Chemistry, Faculty of Natural Sciences, Comenius University, Bratislava, Slovakia; ^5^Axxence Slovakia, s.r.o., Bratislava, Slovakia

**Keywords:** biotransformation, green notes, recombinant protein production and purification, alcohol dehydrogenase, formate dehydrogenase, acetophenone reduction, immobilized enzyme regeneration

## Abstract

The aim of this study was to develop immobilized enzyme systems that reduce carbonyl compounds to their corresponding alcohols. The demand for natural aromas and food additives has been constantly growing in recent years. However, it can no longer be met by extraction and isolation from natural materials. One way to increase the availability of natural aromas is to prepare them by the enzymatic transformation of suitable precursors. Recombinant enzymes are currently being used for this purpose. We investigated *trans-*2-hexenal bioreduction by recombinant *Saccharomyces cerevisiae* alcohol dehydrogenase (ScADH1) with simultaneous NADH regeneration by recombinant *Candida boidinii* formate dehydrogenase (FDH). In a laboratory bioreactor with two immobilized enzymes, 88% of the *trans-*2-hexenal was transformed to *trans-*2-hexenol. The initial substrate concentration was 3.7 mM. The aldehyde destabilized ScADH1 by eluting Zn^2+^ ions from the enzyme. A fed-batch operation was used and the *trans-*2-hexenal concentration was maintained at a low level to limit the negative effect of Zn^2+^ ion elution from the immobilized ScADH1. Another immobilized two-enzyme system was used to reduce acetophenone to (S)-1-phenylethanol. To this end, the recombinant alcohol dehydrogenase (RrADH) from *Rhodococcus ruber* was used. This biocatalytic system converted 61% of the acetophenone to (S)-1-phenylethanol. The initial substrate concentration was 8.3 mM. All enzymes were immobilized by poly-His tag to Ni^2+^, which formed strong but reversible bonds that enabled carrier reuse after the loss of enzyme activity.

## Introduction

In recent years, there has been an increasing demand for natural aromatic chemicals in the food and cosmetics industries. Natural aromas are produced by isolation from natural materials (Sharmeen et al., 2021), biotransformation of natural compounds, and *de novo* production by microorganisms (Shaaban et al., 2016; Paulino et al., 2021). Commercially important compounds are derived from green plant parts and are collectively referred to as “green odor.” Eight volatile compounds including C6-aldehydes and C6-alcohols such as the leaf aldehyde *trans-*2-hexenal and the leaf alcohol *cis*-3-hexenol contribute to “green odor” (Hatanaka, 1996; Poltronieri et al., 2019). The synthesis of “green odor” compounds starts with free polyunsaturated fatty acids (PUFAs) that are transformed by specific lipoxygenases into hydroperoxy fatty acids subsequently cleaved by specific hydroperoxide lyases into aldehyde and oxoacid moieties (Poltronieri et al., 2019). The aldehydes undergo thermal isomerization or may be reduced by dehydrogenases to other C6 products. Hydroperoxide lyase is inhibited by α-, β-unsaturated aldehydes such as *trans*-2-hexenal that bind sulfhydryl groups. These aldehydes are generated by isomerization of non-inhibiting primary products such as hexanal or *cis*-3-hexenal (Suurmeijer et al., 2000; Long et al., 2010). This inhibition is limited by *in situ* reduction of the aldehydes to their corresponding alcohols via alcohol dehydrogenase (ADH) in pure form or in yeast cells. A two-enzyme system consisting of hydroperoxide lyase and ADH cleaved 13-hydroperoxide linolenic acid faster than hydroperoxide lyase alone (Gargouri et al., 2004).

Alcohol dehydrogenases are also used in natural aroma preparation to reduce ketones to secondary alcohols (Ying et al., 2014; Bakonyi et al., 2020). The secondary alcohol 1-phenylethanol has a mild-floral odor and is used extensively in the cosmetic industry (Hawley and Lewis, 1992). Direct isolation of 1-phenylethanol from plants has a low yield and is not cost-effective. However, an alternative approach to 1-phenylethanol generation is enzymatic acetophenone bioreduction (Zhou et al., 2019).

ADH can be used to reduce aldehydes and ketones. Nevertheless, alcohol dehydrogenases differ in terms of structure and substrate specificity. Therefore, suitable ADH must be found for each application. One widely used type of ADH is secreted by *Saccharomyces cerevisiae* (ScADH1). This constitutive enzyme reduces acetaldehyde to ethanol during glucose fermentation (Leskovac et al., 1999). It is usually dimeric in higher eukaryotes but homotetrameric in lower eukaryotes such as yeasts. The latter form has a relatively smaller, narrower active site with a comparatively higher affinity for primary carbonyl compounds. In contrast, the alcohol dehydrogenase from the Gram-positive bacterium *Rhodococcus ruber* (RrADH) reduces ketones and oxidizes secondary alcohols (Hamnevik et al., 2014). RrADH had 5,000-fold higher enzymatic activity on 1-phenylethanol than 2-phenylethanol (Hamnevik et al., 2014). RrADH can bind bulky substrates, as its active site resembles an open cleft. Both proteins are structurally similar NAD(H)-dependent oxidoreductases containing zinc atoms required for their activity. However, RrADH activity is robust at various pH and elevated temperatures. It is also stable in solvents such as acetone used to solubilize hydrophobic ketones and alcohols in the biotransformation. Acetone is also a substrate and co-substrate for certain enzymatic reactions involving nicotinamide cofactor recycling (Karabec et al., 2010; Hamnevik et al., 2014).

Cell-free ADH preparations have high specificity, low product degradation, and superior enzyme activity regulation. However, aldehyde reduction by ADH requires stoichiometric quantities of reduced cofactor. The preparation of certain aromas by aldehyde reduction to alcohols depends on equilibrium reactions. One possible way to improve the efficiency of this process is to shift the reaction equilibrium toward the alcohol product side by increasing the NADH concentration. Therefore, the highest possible NADH concentration should be maintained during the reaction. Nevertheless, these processes are not cost-effective unless reduced cofactors are regenerated. Green aroma chemicals are substantially cheaper than NADH or NADPH. Reduced cofactors may be regenerated enzymatically, electrochemically, or chemically (Weckbecker et al., 2010; Wang et al., 2017). A well-known and highly effective example is the formate dehydrogenase (FDH) reaction using formate as a co-substrate (Shaked and Whitesides, 1980). The equilibrium of the FDH reaction is markedly shifted toward the right because the CO_2_ product is easily removed from the reaction system (Duman et al., 2020). Co-immobilized amine and formate dehydrogenases were used in the continuous production of chiral amine in a packed bed reactor (Franklin et al., 2021). FDH catalysis served as a model for the development of artificial metalloenzymes reducing nicotinamide cofactors resilient to high temperatures, compatible with solvents, and lacking FDH complexity (Basle et al., 2021).

Only a few FDH-based industrial processes are known. Examples include the production of l-*tert*-leucine by reductive amination of α-keto acids with an amino acid dehydrogenase (Evonik Industries AG, Germany) (Bommarius et al., 1998), the reduction of 1-phenyl-2-propanone to 1-phenyl-2-propanole (Forschungszentrum Julich GmbH, Germany), the reduction of 2-oxo-4-phenylbutyric acid to 2-hydroxy-4-phenylbutyric acid (CIBA Spezialitatenchemie AG, Switzerland), and the reduction of 5-(1,3-dioxolan-2-yl)-2-oxopentanoic acid to (S)-2-amino-5-(1,3-dioxolan-2-yl)pentanoic acid (Bristol-Myers Squibb, USA) (Liese et al., 2006). FDH is seldom used in industrial biocatalysis because of its high purchase price. A workaround is FDH overexpression by a suitable host and subsequent enzyme immobilization.

The implementation of enzyme catalysis is often limited by low enzyme thermostability, shear stress sensitivity, and limited use in organic solvents (Chapman et al., 2018). However, the enzymes may be immobilized by binding them to solid supports. The first known attempt was the immobilization of invertase on adsorbent particles. In this manner, the enzyme could be recycled and repeatedly used in catalysis (Nelson and Griffin, 1916). Immobilized enzymes have been extensively investigated and have demonstrated clear advantages over native enzymes. In industrial biocatalysis, enzyme immobilization improves enzyme stability, selectivity, and specificity and reduces inhibition (Mateo et al., 2007; Vasić et al., 2020). This applies to the industrial use of alcohol dehydrogenases which may be facilitated by stabilizing them with suitable immobilization techniques.

The immobilization strategy in this study was based on the use of recombinant poly-His-tag fused enzymes. Several studies showed that this approach enables enzymes to be purified and immobilized on metal chelated supports for subsequent use as biocatalysts (Brena et al., 1994; Beitle and Ataai, 1997; Mateo et al., 2001; Ho et al., 2004). The supports are generally inexpensive and may be prepared from epoxy and iminodiacetic or iminotriacetic acid and subsequent metal cation complexation. This immobilization technique is conducted under gentle conditions compared with direct binding via epoxide groups and eliminates the risk of active site binding to the support. The important advantage of this approach is the possibility to regenerate the biocatalyst by replacing the enzyme at the end of its lifespan. This technique was applied to alcohol dehydrogenases from horse liver (Quaglia et al., 2013), *Kluyveromyces polyspora* (Zhou et al., 2021), and *Aromatoleum aromaticum* (Böhmer et al., 2018). His-tagged ADH from *Thermus thermophilus* was purified and immobilized on a metal support in a single step. In this way, the number of steps required for biocatalyst preparation may be reduced (Kozgus et al., 2014).

This study aimed to develop a biocatalytic redox system based on poly-His-tag fused enzyme immobilization in a flow bioreactor system. Formate dehydrogenase from *Candida boidinii* and alcohol dehydrogenases from *Saccharomyces cerevisiae* and *Rhodococcus ruber* were produced by gene expression in *Escherichia coli* and immobilization on a nickel carrier. The immobilized enzymes were used in two-enzyme biotransformation systems designed for the reduction of trans-2-hexenal and acetophenone to the corresponding alcohols with simultaneous NADH regeneration.

## Materials and Methods

### Chemicals and Reagents

All chemicals were purchased from Sigma-Aldrich Corp. (St. Louis, MO, USA) and Centralchem (Banská Bystrica, Slovakia) unless otherwise stated. *D-*Trehalose was acquired from Apollo Scientific (Stockford, UK). Acetophenone was obtained from Axxence Slovakia Ltd. (Bratislava, Slovakia). Deionized water (RiOs TM water purification system; EMD Millipore, Billerica, MA, USA) was used in all experiments.

### Bacterial Strains and Plasmids

All bacterial strains and plasmids were used in ADH1 production from *Saccharomyces cerevisiae* (Utekal et al., 2014; Levarski et al., 2018) and in FHD production from *Candida boidinii*. Gene expressions and enzyme preparations were described in a previous report (Levarski et al., 2018). Bacterial strains and plasmids used in RrADH production are listed in Tables 1, 2.

**Table 1:** *Escherichia coli* strains used in this study.

**Strain**	**Genotype**	**Reference**
DH5α	F– *ϕ80lacZZ ΔlacZYA-argF) U196 endA1 recA1 hsdR17 (rk–, mk+) supE44 thi-1 gyrA96 relA1 phoA*	Invitrogen (Carlsbad, California, USA)
BL21(DE3)	F– *ompT gal dcm lon hsdSB(rB– mB–) λ(DE3 [lacI lacUV5-T7 gene 1 ind1 sam7 nin5])*	Novagen (Madison, Wisconsin, USA)
C41(DE3)	F–*ompThsdSB (rB– mB–) galdcm(DE3)*	Lucigen (Middleton, Wisconsin, USA)
C43(DE3)	F– *ompT gal dcm hsdS_*B*_(r${}_{B}^{-}$ m${}_{B}^{-}$)(DE3)*	Lucigen (Middleton, Wisconsin, USA)
Rv308ai	Su– lacX74*, gal::ISII(OP308), araBAD::*T7RNAP*, strA*	Krahulec et al., 2010
SHuffle T7®	F' *lac, pro, lacI^*q*^* / Δ(ara-leu)7697 araD139 fhuA2 lacZ::T7 gene1 Δ(phoA)PvuII phoR ahpC* galE (or U) galK λatt::pNEB3-r1-*cDsbC*(Spec^R^, *lacI^*q*^*) Δ*trxB rpsL150*(Str^R^) Δ*gor* Δ*(malF)3*	New England Biolabs (Massachusetts, USA)
LEMO21(DE3)	*fhuA2 [lon] ompT gal (λ DE3) [dcm] ΔhsdS/ pLemo*(Cam^R^) *λ DE3 = λ sBamHIo ΔEcoRI-B int::(lacI::PlacUV5::T7 gene1) i21 Δnin5* pLemo *=* pACYC184*-PrhaBAD-lysY*	New England Biolabs (Massachusetts, USA)

**Table 2:** Plasmids used in this study.

**Plasmid**	**Reference**
pET29b-RrADH	This work
pET29-FDH	Levarski et al., 2018
pRSFDuet-ADH	Utekal et al., 2014
pGro7®	Takara (Kyoto, Japan)

*Rhodococcus ruber sadh* was synthesized, codon-optimized for *Escherichia coli* (GenScript, Piscataway, NJ, USA), and cloned into pET29b via *Nde*I/*Xho*I restriction sites. The recombinant plasmid pET29-RrADH was sequenced to confirm the correct sequences and open reading frames (ORFs).

### RrADH Plasmid Transformation

Bacterial strains with chemically induced competence were transformed by the pET29-RrADH plasmid (Hanahan, 1983). Briefly, overnight bacterial cultures were incubated at 37°C in Luria-Bertani (LB) medium without antibiotic until OD_600_ = 0.5–0.6. The cells were centrifuged for 5 min. at 1,920 × *g*. The bacterial pellet was resuspended in the buffer according to the protocol and kept on ice for 1.5 h. Glycerol (15% v/v) was added to competent cell aliquots and the suspensions were either used in *E. coli* transformation or stored at −70°C until the subsequent experiments.

### RrADH Gene Expression

*Escherichia coli* cells containing the pET-RrADH were grown in 20 mL LB medium (10 g/L peptone, 5 g/L yeast extract, and 5 g/L NaCl), Terrific-Broth medium (TB) (24 g/L yeast extract, 20 g/L peptone, 40 g/L glycerol, 0.017 M KH_2_PO_4_, and 0.072 M K_2_HPO_4_) and Dynamite medium (24 g/L yeast extract, 20 g/L peptone, 40 g/L glycerol, 1 mM L MgSO_4_, 0.017 M KH_2_PO_4_, and 0.072 M K_2_HPO_4_) in Erlenmeyer flasks containing 1% glucose (w/v) and 1 mM kanamycin and incubated overnight. Then 0.5 mL culture was transferred to 50 mL fresh medium supplemented with 1% (w/v) glucose. The culture was shaken at 160 rpm on a Multitron Standard orbital shaker (Infors HT GmBH, Bottmingen, Switzerland) at 20°C, 28°C, or 37°C until exponential growth (OD_600_ range 0.5–0.8). RrADH was induced by adding isopropyl-β-d-1-thiogalactopyranosid (IPTG) to a final concentration of 1 M. Large-scale expression was performed in 1 L medium plus 0.1% glucose (w/v) inoculated with 100 mL overnight culture in a Biostat B plus-2L MO bioreactor (Sartorius AG, Göttingen, Germany). Bioreactor aeration and initial stirring were set to 0.6 vvm and 200 rpm, respectively. In response to the metabolic activity of the cells, the O_2_ concentration declined to 2% saturation and was maintained by agitation at 200–2,000 rpm. Samples were taken before and periodically after induction and analyzed by 12% SDS-PAGE electrophoresis (Laemmli, 1970). The cells were incubated for 18 h after induction.

### RrADH Purification

An ultrasonic homogenizer (Sonopuls HD3200; Bandelin Electronic GmbH, Berlin, Germany) was fitted with a KE76 probe and used to disrupt the cells. To increase RrADH solubility during sonication, the effect of adding 0.7 M *D-*trehalose to the sonication buffer was analyzed. The bacterial pellet was kept on ice, resuspended in 30 mL sonication buffer (0.5 M NaCl, 50 mM Tris HCl (pH 8), and 0.7 M *D-*trehalose) and disrupted by 10–15 cycles of 30 s sonication and 30 s breaks. The lysate was centrifuged at 7,690 × *g* for 15 min. A supernatant (soluble fraction) sample was removed for PAGE analysis. The pellet (insoluble fraction) was resuspended in sonication buffer plus 2% SDS (w/v), shaken for 1 h, and centrifuged at 7,690 × *g* for 1–2 h. The supernatant fractions were pooled and their soluble protein content was quantified. RrADH was purified by IMAC affinity chromatography in an ÄKTA Avant 25 system (GE Healthcare, Little Chalfont, UK) with a 5-mL HisTrap FF column (GE Healthcare, Little Chalfont, UK) equilibrated by Ni^2+^ ions. The disrupted cell mixture was centrifuged at 7,690 × *g* and 4°C for 2 × 30 min to remove cell debris. The HisTrap FF column was washed with 2–5 column volumes (CV) water and 2–5 CV equilibration buffer (50 mM Tris HCl (pH 8) and 0.5 M NaCl). The samples were directly loaded onto the column at 0.1–1 CV/min pump speed. The column was washed with 10–15 CV equilibration buffer to remove non-specifically bound material. Then 2–5 CV elution buffer (50 mM Tris HCl (pH 8), 0.5 M NaCl, and 0.5 M imidazole) was used to elute the RrADH. Samples were drawn by an automatic fraction collector and analyzed by SDS-PAGE. GelAnalyzer v. 19.1 (Lazar et al.) was used in densitometric analysis to determine RrADH expression and/or its soluble:insoluble ratio during purification.

The eluted purified protein was mixed with glycerol to a final concentration of 50% (v/v) and stored at −20 °C. The pooled elution fractions were dialyzed against storage buffer (10 mM Tris HCl (pH 8), 100 mM NaCl, and 50% glycerol (v/v) using a 10 kDa MW cutoff membrane. Protein concentration was measured by the Bradford assay (Bradford, 1976).

### Enzyme Immobilization

Enzymes were immobilized according to a previously described method (Mateo et al., 2001). Eupergit-CM (100 mg) was mixed with 2 mL of a solution containing 0.1 M boric acid and 2 M iminodiacetic acid. The solution pH was adjusted to 8.5 with 5 M NaOH. The solution was placed in a sealed dark vial and shaken at 200 rpm on an orbital shaker at room temperature. After 2 h, the support was washed with an excess of deionized water, mixed with 2 mL of 0.05 M sodium phosphate buffer solution (pH 7.0) containing 0.1 M NiCl_2_ and 1 M NaCl, and agitated at 200 rpm and room temperature for 2 h. The Ni^2+^ carrier was washed with deionized water and used in enzyme immobilization.

The Ni^2+^ carrier was quantitatively transferred from the frit to a vial containing 1 mL alcohol dehydrogenase solution, 10 mM Tris HCl (pH 8), 100 mM NaCl, 50% glycerol (v/v), and 2 mL of 0.1 M phosphate buffer (pH 7.4) and shaken at 200 rpm and room temperature for 24 h. The immobilized enzyme preparation was filtered on a frit, washed with 0.1 M phosphate buffer (pH 7.4), and stored in it. The Bradford assay confirmed that in the case of ScADH1 99% of the protein was bound to the carrier and the protein content on the carrier was 25 mg/g. The activity measurement showed a 20.0% activity yield relative to the total ADH activity in the immobilization solution. The immobilization yield of RrADH was lower due to the lower purity of the enzyme preparation and it was 30 % providing the protein content on the carrier of 10 mg/g and the ADH activity yield of 20% relative to the enzyme activity in the solution used for the immobilization.

The immobilization of format dehydrogenase was carried out in the same way. Two milliliters of the enzyme solution (in 10 mmol/L Tris.HCl pH 8, 100 mmol/L NaCl, 50 % glycerol) were used undiluted. The immobilized enzyme preparation was then washed with 0.1 M phosphate buffer (pH 8) and stored in it. The Bradford assay confirmed that 83% of the protein was bound to the carrier and the protein content on the carrier was 20 mg/g. The activity measurement showed a 21.1% activity yield relative to the total FDH activity in the immobilization solution.

The immobilized enzyme preparations were packed in individual small peek columns (2.1 mm i.d. × 50 mm length; VICI AG International, Schenkon, Switzerland) and used in the subsequent activity and biotransformation measurements.

### RrADH and FDH Activity

RrADH and FDH activity levels were determined by continuous spectrophotometry at 340 nm and 25°C. Enzymatic assays were performed in 1 mL reaction mixtures. To determine the oxidation activity of RrADH, isopropanol was the substrate and NAD was the cofactor. The reagent concentrations were 100 mM isopropanol, 100 mM sodium phosphate buffer (pH 7.0–8.0 in 0.2-unit increments), 1.5 mM NAD, and 0.9 μg/mL RrADH. The reduction reaction system consisted of 100 mM Na^+^ phosphate buffer (pH 7.0–8.0 in 0.2-unit increments), 100 mM acetone, 0.16 mM NADH, and 0.9 μg/mL RrADH. Absorbance at OD_340_ corresponding to the NADH concentration was monitored for 5 min. One unit (1 U) RrADH activity was defined as the amount of enzyme converting 1 μmol isopropanol to acetone in 1 min in the presence of NAD at pH 7.2 and 25°C.

FDH activity was determined under the same conditions as that for RrADH except the reaction mixture comprised 100 mM Na^+^ phosphate buffer (pH 8), 100 mM sodium formate, 1.5 mM NAD, and 5.84 μg/mL FDH. One unit (1 U) FDH was defined as the amount of enzyme converting 1 μmol formate to CO_2_ in 1 min in the presence of NAD at pH 7 and 37°C (Bergmeyer, 1974).

### Biotransformation of Acetophenone to 1-Phenylethanol in a One-Pot Reaction System

The biotransformation was conducted in a 1 mL reaction volume and the mixture consisted of 100 mM Na^+^ phosphate buffer (pH 7.2), 100 mM sodium formate, 1.5 mM NAD, 8.33 mM acetophenone, 0.9/9 μg/mL RrADH (in 1 μL and 10 μL solution), and 5.84/58.4 μg/mL FDH (in 1 μL and 10 μL solution). The mixture containing all reagents except RrADH was incubated in a Multitron Standard shaker (Infors HT GmBH, Bottmingen, Switzerland) at 30°C for 30 min allowing the FDH to reduce NAD to NADH. RrADH was then added and the 50 μL samples were regularly removed. The samples were diluted in 200 μL acetone to denature the enzymes and analyzed by gas chromatography as described below.

### Investigation of Immobilized ScADH1 Properties

For testing the ScADH1 activity a substrate solution was used, containing 0.1 mol/L phosphate buffer solution of pH 7.4 with 0.17 mmol/L of NADH and 4 mmol/L of trans-2-hexenal. Alternatively, the substrate solution contained the same buffer and 1.7 mmol/L of NAD and 25 mmol/L of ethanol.

The immobilized enzyme column was placed in a water bath at 30°C and the substrate solution was pumped through the column with a peristaltic pump at a flow rate of 0.9 mL/min. Samples were taken at the column output and NADH concentrations were measured by spectrophotometry at 340 nm. A molar extinction coefficient of 6.22/cm/mM was used to calculate the NADH concentration.

### Investigation of Biotransformations Catalyzed by Immobilized ADH/FDH

**Figure 1:**
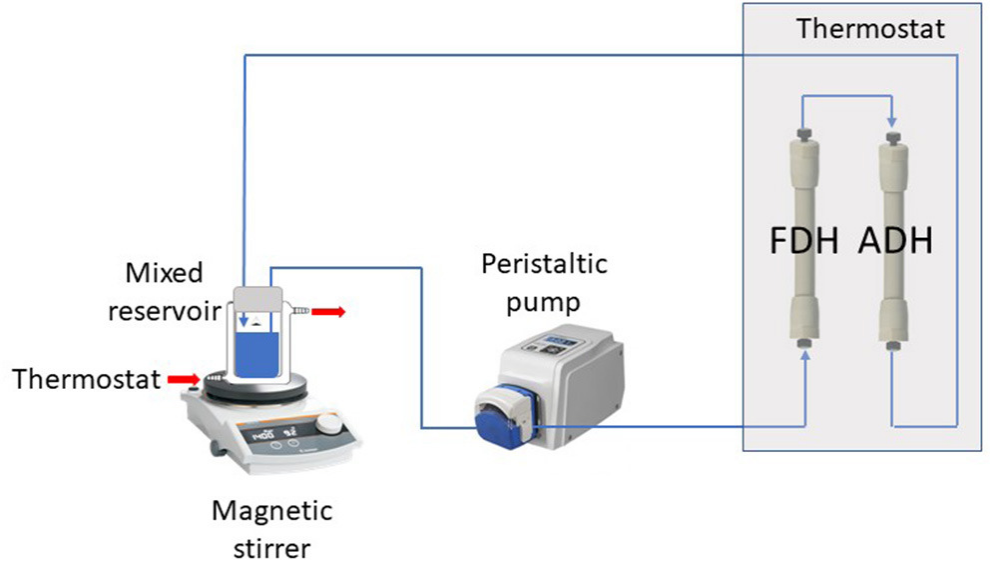
Experimental two-enzyme system with infinite recirculation.

ScADH1-catalyzed bioreduction of *trans-*2-hexenal to *trans-*2-hexenol was examined in an infinite recirculation system (Figure 1). The system was fitted with two separate columns containing immobilized ADH and FDH and immersed in a water bath at 30°C. The total void volume of the columns with connection capillaries was 2 mL. The stirred glass-jacketed vessel initially contained 18 mL of 0.1 M phosphate buffer (pH 7.4), 18 mM sodium formate, *trans-*2-hexenal at various concentrations, or other substances. The solution was pumped by a peristaltic pump through the immobilized enzyme columns at a flow rate of 2 mL/min and returned to the stirred vessel. After flow rate and temperature stabilization, the reaction was initiated by adding 2 mL of 17 mM NAD. During the reaction, samples were withdrawn from the vessel and analyzed for NADH, *trans-*2-hexenal, and *trans-*2-hexenol concentrations.

The procedure described previously was applied for acetophenone reduction with RrADH. The initial acetophenone concentration was 8.33 mM or 1 g/L.

### Gas Chromatography

Before the analysis, reaction mixture samples were diluted fourfold with acetone (Chromasolv for GC; Sigma-Aldrich Corp., St. Louis, MO, USA). Gas chromatography (GC) measurements were performed on a 6890N gas chromatograph with a flame ionization detector (FID) (Agilent Technologies, Santa Clara, CA, USA). The injection port was maintained at 260°C and a 1-μL sample was injected at a 10:1 split ratio. The samples were separated with a 30 m (L) × 0.25 mm (ID) capillary column coated with a 0.25-μm film (df) of 5% phenylmethylpolysiloxane. The stationary phase was HP-5 (Agilent Technologies, Santa Clara, CA, USA). The column temperature was 80°C for the first min, increased to 130°C at a ramp of 7°C/min, and held at 130°C for 2 min. The carrier gas was He and the flow rate was 1.9 mL/min. The FID temperature was 250°C. Data were processed with Agilent ChemStation v. B.04.03. (Agilent Technologies, Santa Clara, CA, USA).

### Enantioselective Gas Chromatography

The enantiomeric purity of the 1-phenylethanol was determined by GC using CYCLOSIL-B (Agilent Technologies, Santa Clara, CA, USA) fitted with a chiral capillary column (30 m (L) × 0.25 mm (ID) × 0.25 μm df). One microliter sample was injected at a 10:1 split ratio. The carrier gas was He and the flow rate was 2.7 mL/min. The column temperature range was 80–240°C and the ramp was 15°C/min. The injector and FID temperatures were 260°C and 250°C, respectively.

### Determination of Zn^2+^ Release From ADH

To establish Zn^2+^ cation release from the immobilized ADH, the Zn^2+^ ion concentration in the column eluent was determined by complexation with dithizone (Song et al., 1976). Fifty milligrams of the carrier with immobilized ADH were placed in two vials containing 1 mL of 0.1 M phosphate buffer (pH 7.4). One vial contained 1.6 mg/mL *trans-*2-hexenal. The vials were gently agitated for 30 min. The immobilized enzyme particles were removed by filtration and 100 mL of 1 mg/mL dithizone in 0.1 M NaOH was added to each sample. The samples were measured spectrophotometrically at 555 nm against a blank containing phosphate buffer solution mixed with dithizone solution. The zinc concentration was interpolated from a standard curve plotted using various Zn^2+^ concentrations.

## Results

### RrADH Expression and Purification

**Figure 2:**
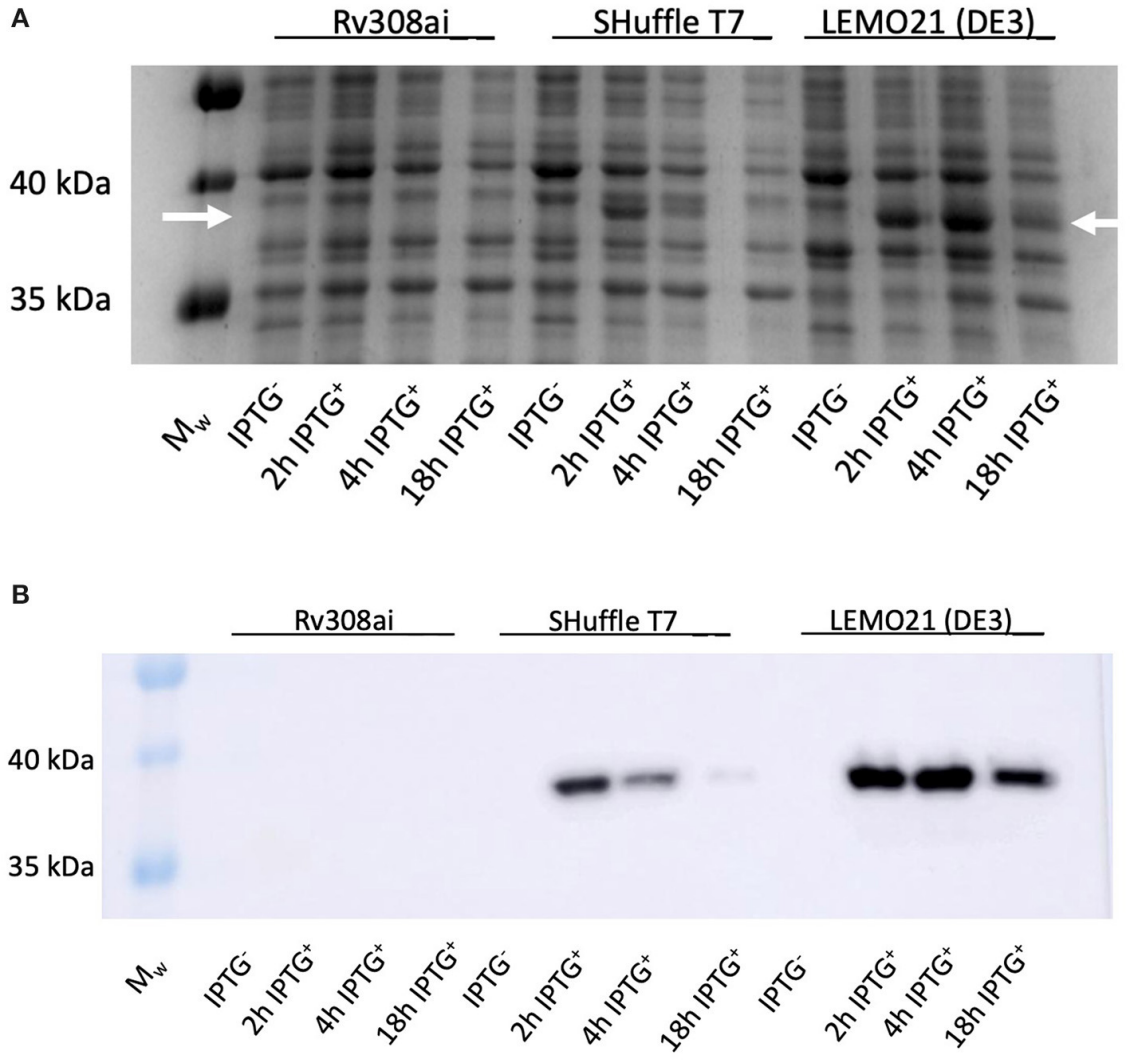
RrADH 50 mL expression in E. coli strains Rv308ai, Shuffle T7, LEMO21 (DE3) in TB medium at 37°C analyzed by SDS-PAGE **(A)** and Western Blot **(B)**. M_w_ – molecular weight marker; IPTG ^−^ - non-induced expression; 2 h IPTG ^+^ - 2 h after induction; 4 h IPTG ^+^ - 4 h after induction; 18 h IPTG ^+^ - 18 h after induction (arrows indicate the approximate position of RrADH).

**Figure 3:**
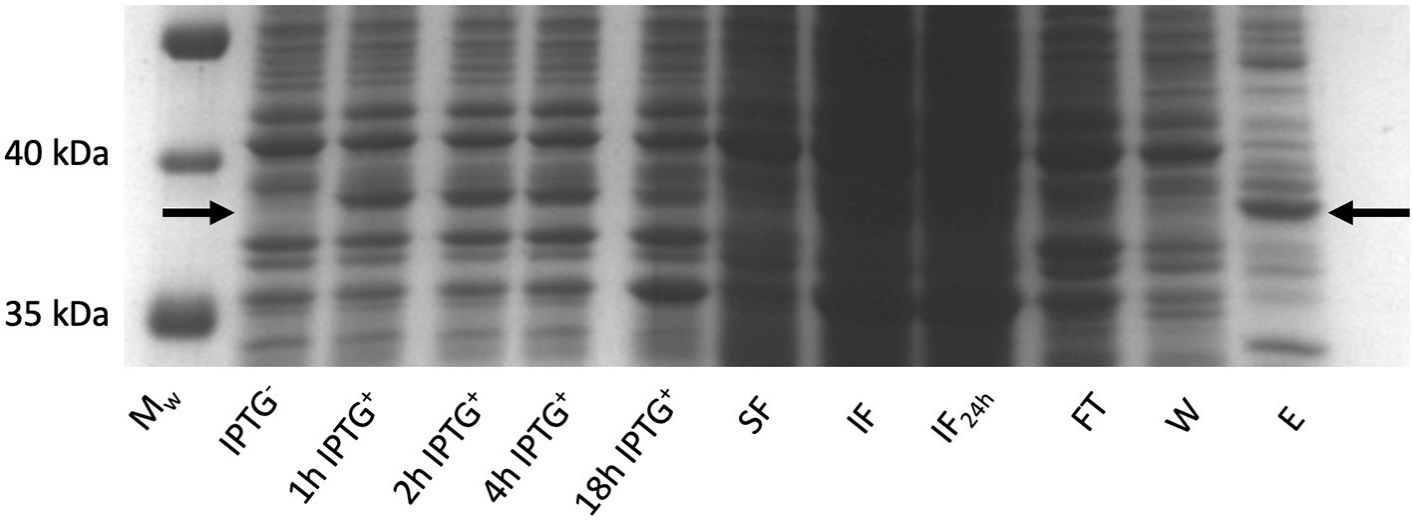
RrADH expression in *E. coli* LEMO21 (DE3) strain in 1 liter (bioreactor) of TB medium at 28°C analyzed by SDS-PAGE. M_w_ – protein ladder; IPTG ^−^ - before induction with IPTG; 1 h IPTG ^+^ - 1 h after induction; 2 h IPTG ^+^ - 2 h after induction; 4 h IPTG ^+^ - 4 h after induction; 18 h IPTG ^+^ - 18 h after induction; SF – soluble fraction; IF – insoluble fraction; IF24 – insoluble fraction incubated in sonication buffer with addition of 2 % SDS (w/v) for 24 h; FT – IMAC flow-through; W – IMAC wash; E – IMAC elution fraction.

*Rhodococcus ruber sadh* was expressed in the *E. coli* expression strains BL21(DE3), C41(DE3), C43 (DE3), Rv308ai, SHuffle T7®, and LEMO21(DE3). The highest *sadh* expression level ( ≤ 15% of total soluble proteins) was determined for LEMO21(DE3) incubated in TB medium at 28°C according to the SDS-PAGE analysis (Figure 2A) and western blot (Figure 2B). However, most of the protein forming the homotetramer (MW 144.77 kDa) was expressed in inclusion bodies and was, therefore, inactive. After adding *D-*trehalose to the sonication solution, the soluble fraction markedly increased (Figure 3). Thus, 45–50 mg purified protein was obtained from 1 L culture medium and purity was increased 2.5 × . Subsequent co-expression experiments with chaperone GroEL and 8 M urea during purification did not dramatically improve protein solubility (data not shown).

### RhADH pH-Activity Profile

**Figure 4:**
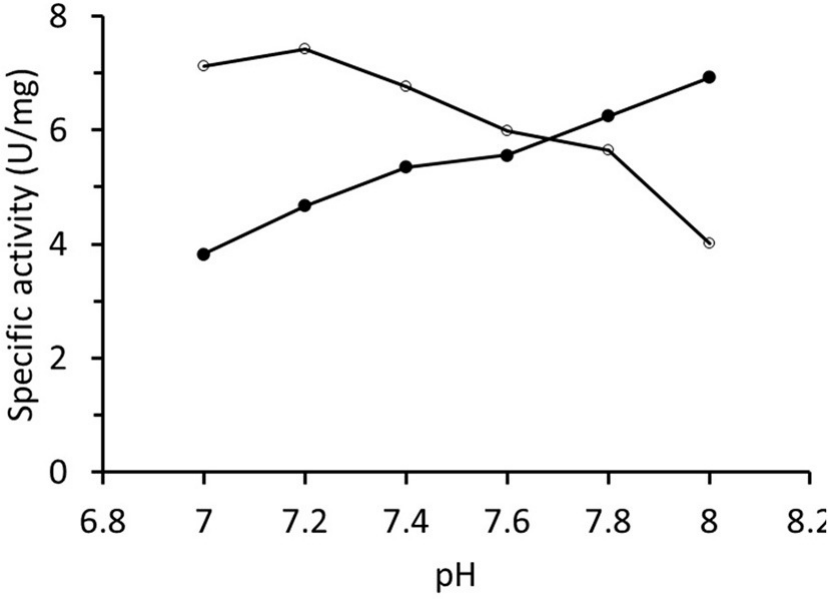
pH-activity profile of recombinant alcohol dehydrogenase from *Rhodococcus ruber* determined for isopropanol oxidation (∙) and acetone reduction (°).

The specific activity of RrADH during purification and in the final preparations was determined by measuring isopropanol oxidation. The rate was always in the range of 1–8 U/mg. The final enzyme preparation was characterized in terms of the pH-activity profile that increased in the examined interval of pH values (Figure 4). The pH-activity profile for acetophenone reduction catalyzed by RrADH exhibited the opposite trend with a sight maximum at pH ~7.2. Hence, pH 7.2 was selected for the preliminary trials on acetophenone bioreduction under soluble enzyme conditions.

### Acetophenone Biotransformation to 1-Phenylethanol in a One-Pot Reaction

**Figure 5:**
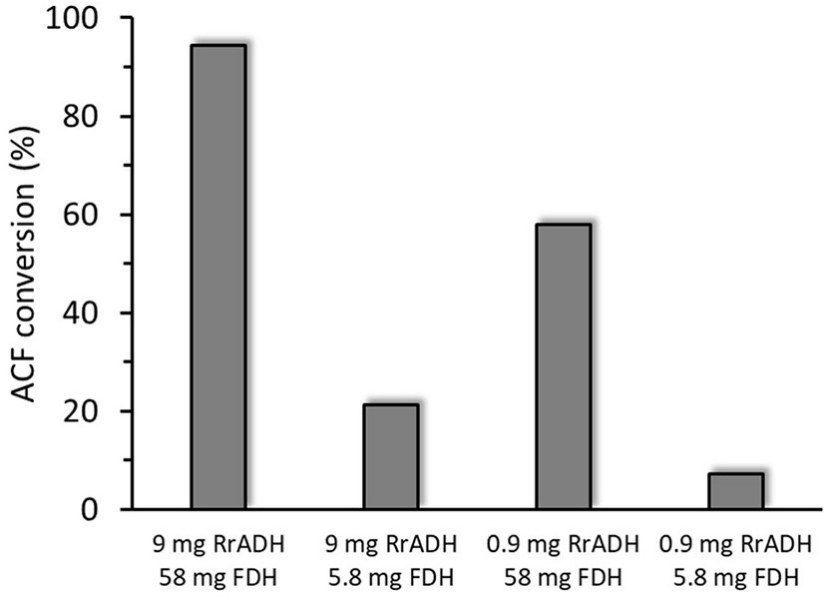
Bioreduction of ACF to 1-phenylethanol in soluble RrADH and FDH system at variable amounts of enzyme in 1 mL of the reaction mixture.

**Figure 6:**
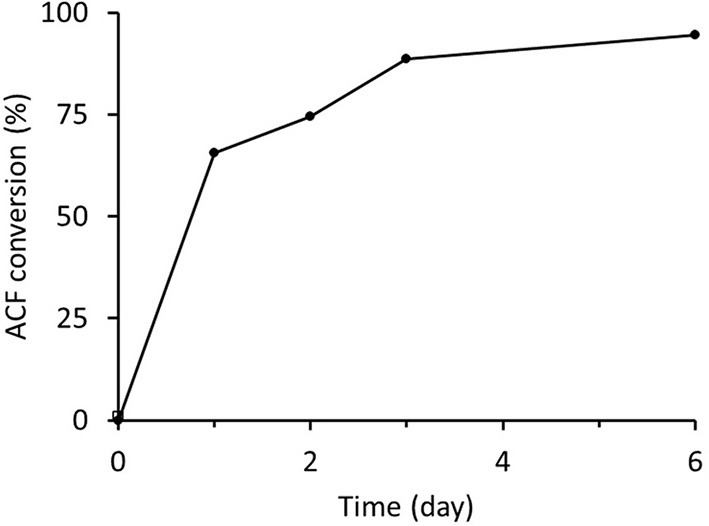
Bioreduction of ACF to 1-phenylethanol in soluble RrADH and FDH system.

Freshly prepared RrADH was used for bio-reduction of acetophenone to 1-phenylethanol. The reaction catalyzed by recombinant RrADH was coupled with NADH regeneration by recombinant formate dehydrogenase reaction. Four different enzyme concentrations were tested to identify the conditions required for maximum acetophenone reduction to 1-phenylethanol. According to the results shown in Figure 5, under optimum conditions ACF conversion was >90% whereas it was <10% at suboptimal enzyme concentrations. The reaction kinetics at the optimal enzyme concentrations (9 μg/mL RrADH and 58.4 μg/mL FDH) are shown in Figure 6. The reaction course was followed for 6 days. In the first 3 days, ACF conversion was nearly 90%. RrADH had very high specificity; enantiomeric GC revealed that under all reaction conditions, RrADH produced 100% S-1-phenylethanol.

### Immobilized ScADH1 Properties

**Figure 7:**
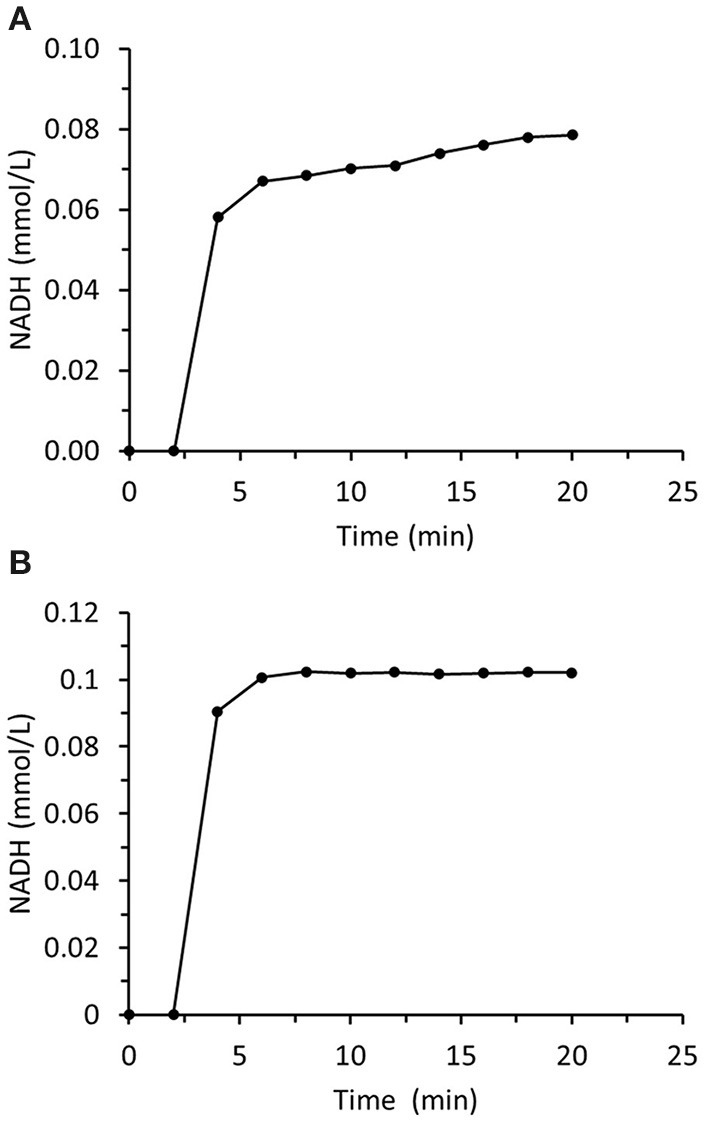
Time dependence of NADH concentration in the output of the column packed with immobilized ScADH1. The column was fed with the substrate solution containing **(A)** −0.17 mmol/L of NADH and 4 mmol/L of *trans*-2-hexenal; **(B)** −1.7 mmol/L of NAD and 25 mmol/L of ethanol.

The principal aim of this study was to prepare and immobilize recombinant enzymes to construct two-enzyme biocatalytic systems that reduce aliphatic and aromatic carbonyl compounds to their corresponding alcohols. The first system was based on ADH from *Saccharomyces cerevisiae* known as ScADH1 that is suitable for aliphatic aldehyde reduction. It is a homotetramer with a total molecular weight of 149.54 kDa. Each monomer is 36.84 kDa. Experiments on immobilized ScADH1 showed that operational stability was a critical point in *trans-*2-hexenal reduction (Figure 7A). A breakthrough in NADH concentration was measured at the output of the small column packed with immobilized ScADH1 after switching from buffer to reaction mixture containing *trans-*2-hexenal and NADH. The concentration of the latter was measured up to quasi-steady-state conditions. Nevertheless, the actual steady state was never reached. The output NADH concentration progressively increased as its consumption rate declined in response to the loss of enzyme activity. In contrast, when ScADH1 was used to catalyze ethanol oxidation, steady-state output NADH concentration was reached (Figure 7B).

**Figure 8:**
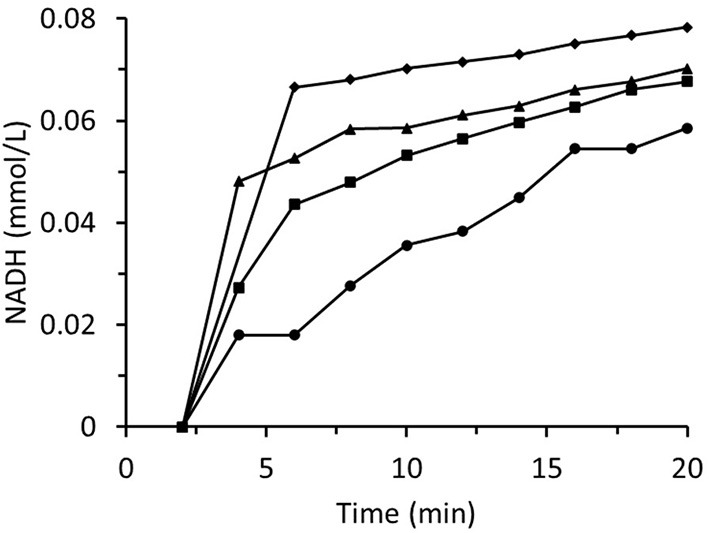
Time dependences of output NADH concentration for the column packed with immobilized ScADH1 and fed with 0.17 mmol/L of NADH solution containing variable inlet *trans*-2-hexenal concentrations (mg/mL): ♦ - 0.2; ▴ - 0.4; ■ - 0.8; ∙ - 1.6.

**Figure 9:**
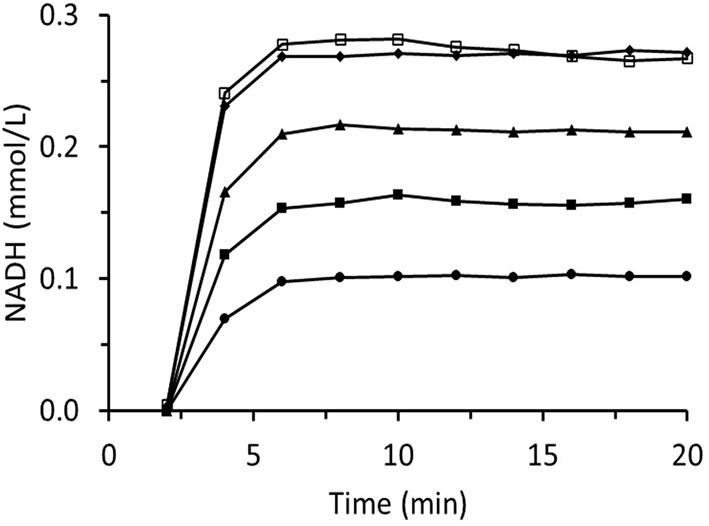
Time dependences of output NADH concentration for the column packed with immobilized ScADH1 and fed with 0.17 mmol/L of NADH solution containing variable inlet *trans*-2-hexenol concentrations (mg/mL): ♦ - 0.2; ▴ - 0.4; ■ - 0.8; ∙ - 1.6; □ - 3.2.

Figure 8 summarizes data for experiments wherein the inlet *trans-*2-hexenal concentration was varied. The rate of loss of enzyme activity increased with *trans-*2-hexenal concentration. An analogous experiment involving *trans-*2-hexenol oxidation presented no enzyme activity loss (Figure 9).

These results could be explained by the role of Zn^2+^ cations in the enzyme structure (Hao and Maret, 2006). The reactions between the enzyme Cys residues and the aldehyde groups broke the bonds between the enzyme and zinc cations. As Zn^2+^ is a part of the active site of ScADH1, the reactions would automatically inactivate the enzyme. This hypothesis was tested by incubating immobilized ScADH1 in 1.6 mg/mL *trans-*2-hexenal. Then, the release of Zn^2+^ ions into the surrounding solution was analyzed. The zinc concentration was 2.4 mg/mL which corresponded to 67% of the amount of zinc originally bound to the enzyme molecules. Therefore, the aldehyde concentration in the reaction system must be minimized during biocatalysis.

### *Trans-*2-Hexenol Production in the Immobilized Two-Enzyme System

**Figure 10:**
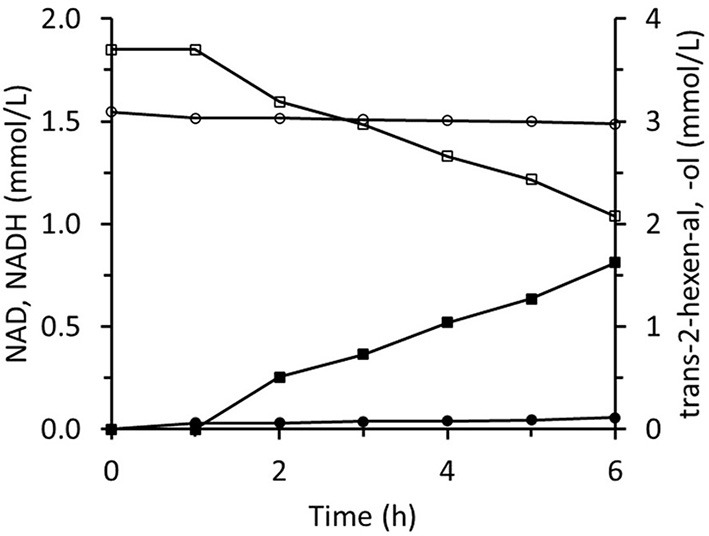
Reaction course in the infinite recycling system with FDA and ADH: ■ - *trans*-2-hexenol; □ - trans-2-hexenal; ∙ - NADH; ° - NAD.

**Figure 11:**
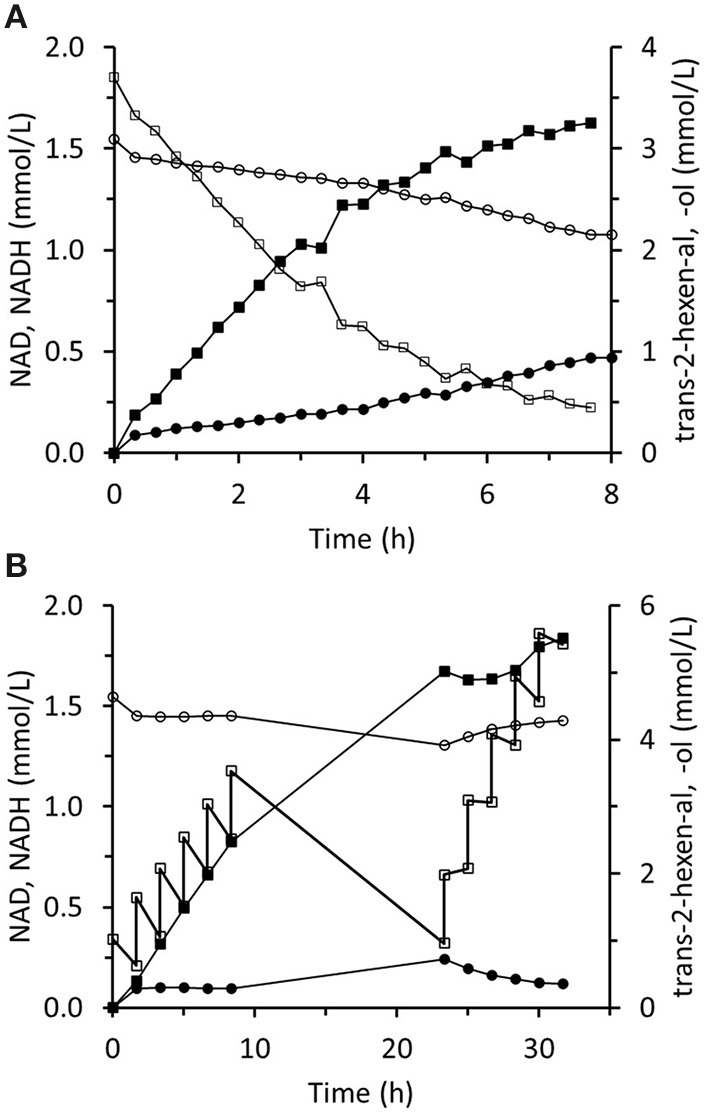
Reaction course in the infinite recycling system with increased FDH amount under batch operation **(A)** and fed-batch operation **(B)**. ■ - *trans*-2-hexenol; □ - *trans*-2-hexenal; ∙ - NADH; ° - NAD.

The immobilized two-enzyme biocatalytic system for *trans-*2-hexenal reduction is illustrated in Figure 10. The NADH concentration was constantly low, which means that the limiting factor was FDH activity. In an experiment using sixfold greater FDH activity, higher NADH concentrations were reached and the *trans-*2-hexenol production rate was enhanced (Figure 11A). In this case, 88% conversion and a final 3.4 mM *trans-*2-hexenol concentration were achieved after 8 h. A fed-batch operation was also tested. The *trans-*2-hexenal was added incrementally to keep its concentration low and limit the effect of zinc cation elution from the immobilized ScADH1. A maximum *trans-*2-hexenol concentration of 5.5 mM (0.55 mg/mL) was attained by sequential addition of *trans-*2-hexenal (Figure 11B).

### 1-Phenylethanol Production in an Immobilized Two-Enzyme System

**Figure 12:**
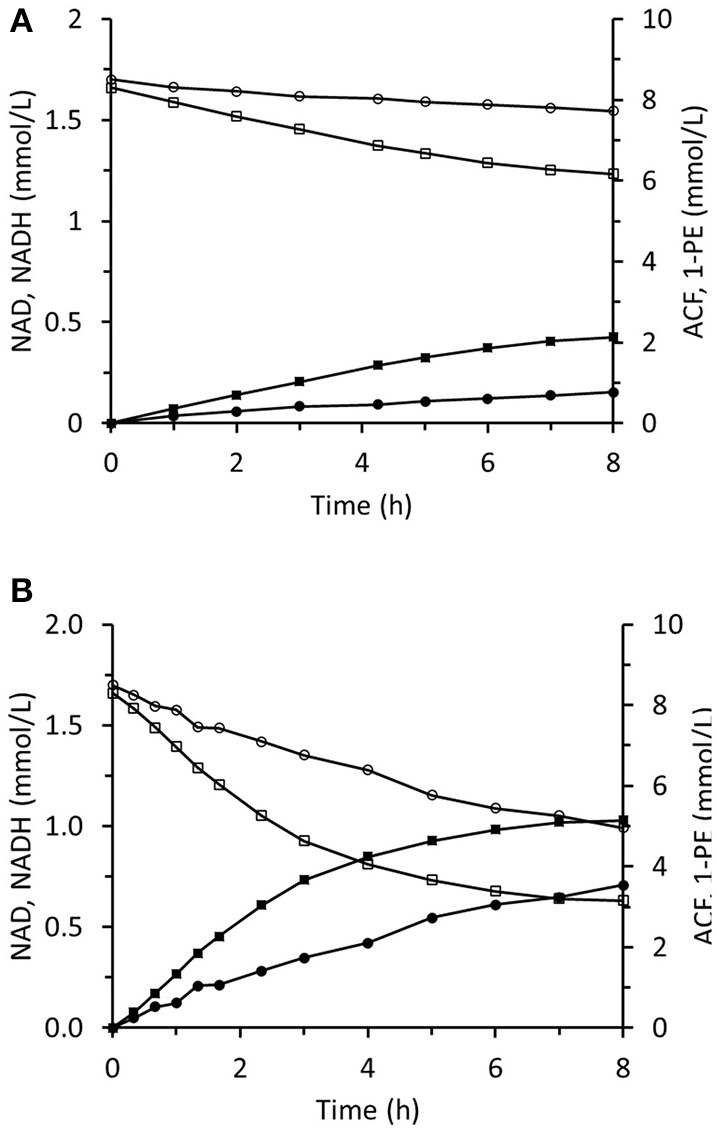
Reaction course in the infinite recycling system with FDA and RrADH: ■ - 1-phenylethanol; □ - acetophenone; ∙ - NADH; ° - NAD. The volume of reaction solution in mixed reservoir was 18 mL **(A)** and 6 mL **(B)**.

The method of ScADH1-catalyzed *trans-*2-hexenal bioreduction was applied to acetophenone bioreduction using immobilized RrADH. The required NADH was regenerated during biotransformation via coupled formate oxidation catalyzed by immobilized FDH. Biotransformation in the infinite recirculation system during the first 8 h of reaction is depicted in Figure 12A. The 1-phenylethanol concentration reached 5.1 mM and corresponded to an ACF conversion of 61% and remained constant during the following 18 h. The experiment was repeated using the same immobilized enzymes and virtually the same results were obtained. This shows that the immobilized enzymes were stable, and the reaction was stopped by reaching equilibrium conditions. The immobilized enzyme preparations were regenerated by replacing the spent enzymes with fresh ones (Figure 12B). Immobilized RrADH and FDH were desorbed by pumping 1 M imidazole through packed columns. Enzyme solutions of the foregoing compositions and concentrations were immobilized on the carriers by recirculation through the columns for 24 h. The reaction course was tested, however, in this case with a three-fold reduced volume of the reaction mixture in a stirred tank to accelerate bioreduction. Figure 12B shows that this adjustment considerably increased the reaction rate, but the resulting ACF conversion remained at 61%.

## Discussion

Numerous enzymes of plant, microbial, and animal origin are used to prepare natural flavors by biocatalysis. The enzymes are classified according to the types of reactions they catalyze. Pertinent examples include oxidations and reductions of oxygen-bearing functional groups catalyzed by alcohol dehydrogenases (Paulino et al., 2021). There is a wide diversity of natural aromas bearing functional groups with different reactivities and they are used in industry. Bioreduction and bio-oxidation often require various types of alcohol dehydrogenases. However, alcohol dehydrogenase application in industrial production may be limited by high costs and low stability (Hummel, 1997). Hence, this study aimed to identify suitable alcohol dehydrogenases and economically efficient ways to prepare them by recombinant techniques. Here, we successfully prepared recombinant enzymes and applied them in two important bioreduction reactions. We used them to reduce the aliphatic unsaturated aldehyde *trans*-2-hexenal and the aromatic ketone acetophenone.

The main goal of expressing a biologically active protein is to achieve a high product yield. The *E. coli* system is usually the first choice in this objective as it can be easily manipulated, inexpensively cultured, and rapidly propagated. However, most *E. coli* strains only reach their highest production efficiency after several optimization steps. Therefore, a combination of maximum cell density and soluble protein production is the best way to obtain a high protein yield.

Alcohol dehydrogenase from *Rhodococcus ruber* was expressed in six different *E. coli* strains cultured in Luria-Bertani medium (LB), Terrific broth (TB), or Dynamite medium and at 20°C, 28°C, or 37°C. The LB medium differed from the other two in that it lacked glycerol and had a unique salt content. Ideal results were obtained with the BL21(DE3) and LEMO21(DE3) strains grown in TB medium at 28°C as previously described (Utekal et al., 2014; Levarski et al., 2018). Maximum RrADH expression with respect to soluble fraction content was detected 18 h after induction in LEMO21(DE3) (Figure 2). The addition of 0.7 M *D*-trehalose during cell homogenization improved protein solubility *in vivo* as previously described (Leibly et al., 2012). The yield of purified RrADH was 0.9 mg/mL after one-step IMAC chromatography and was determined by Bradford assay and SDS-PAGE densitometric analysis. Thus, enough enzymes could be prepared for the biotransformation experiments.

A kinetic analysis of native purified RrADH with isopropanol substrate indicated low specific activity (maximum 8 U/mg). Comparison of the pH-activity profiles of opposing reactions (oxidation/reduction) showed substantially different pH dependencies, namely, increasing for isopropanol oxidation and decreasing for acetone reduction (Figure 4). This observation was consistent with the pH-activity profiles for (S)-1-phenylethanol oxidation and acetophenone reduction reported for wild type ADH from *Rhodococcus ruber* (Hamnevik et al., 2014) and recombinant ADH from *Rhodococcus erythropolis* (Kasprzak et al., 2016). This behavior is not exceptional in ADH-catalyzed reactions. Markedly different pH profiles were observed for oxidation and reduction reactions catalyzed by Zn-dependent ADH from *Chloroflexus aurantiacus* (Loderer et al., 2018) and *Aeropyrum pernix* (Hirakawa et al., 2004).

The enzymes prepared herein were successfully tested in a one-pot reaction system for the bioreduction of acetophenone to 1-phenyletanol. There was nearly 95% acetophenone conversion to 1-phenylethanol in 6 days (Figure 5) and 100% of the product was the desired S-1-phenylethanol. Hence, this finding lays the foundation for the design and optimization of immobilized enzymatic biocatalysis systems.

The bioreduction capacity of an immobilized two-enzyme system comprising recombinant poly-His tagged *Saccharomyces cerevisiae* ADH1 and *Candida boidinii* FDH was tested using *trans*-2-hexenal as a substrate in a flow system with infinite recirculation (Figure 1). A similar experimental configuration was designed and tested for bioreduction and simultaneous cofactor regeneration (Zhou et al., 2021) using two enzymes co-immobilized on a packed bed column. The results of the present study demonstrated that co-immobilization is suboptimal when one of the enzymes is substantially less stable and must be replaced more frequently than the other. The aldehyde in the reaction medium diminished ScADH1 activity possibly because of the elution of Zn^2+^ ions vital to the enzyme (Yang and Zhou, 2001). The foregoing observations and the low stability of yeast ADH (Magonet et al., 1992; Leskovac et al., 2002; Bolivar et al., 2012) indicate that immobilized ADH must be regenerated more frequently than immobilized FDH. The use of two enzymes in separate columns enabled optimization of the biotransformation system by increasing the amount of immobilized FDH that limited overall bioreduction because of slow NADH regeneration. According to an earlier study (Zhou et al., 2021), adding an adsorption column for *in situ* product removal (ISPR) could efficiently shift the reaction equilibrium and increase *trans*-2-hexenol production. Yeast alcohol dehydrogenases effectively catalyze transformations of short-chain primary aliphatic alcohols and aldehydes (Dickinson and Monger, 1973; Green et al., 1993). However, they are relatively less effective on secondary alcohols and ketones when they have neighboring alkyl groups larger than the methyl group and are, therefore, sterically hindered (Dickinson and Dalziel, 1967). Considering this substrate specificity, the recombinant poly-His tagged ADH from *Rhodococcus ruber* was chosen instead of yeast ADH for the bioreduction of acetophenone to (S)-1-phenylethanol. Tests on both systems showed that the final bioreduction process did not fully consume the substrate and the conversion was <100%. To ameliorate system performance, future work must investigate the equilibrium and product inhibition phenomena that limit biotransformation. It is also necessary to identify the various pH optima for the oxidation and reduction reactions. Our studies on alcohol dehydrogenase from *Rhodococcus* (Figure 4) corroborate findings reported earlier (Hamnevik et al., 2014; Kasprzak et al., 2016). Bioreduction process improvement may be achieved by implementing hybrid systems combining reaction with *in situ* product removal, as they are promising ways to intensify biocatalytic processes (Schügerl and Hubbuch, 2005) (Woodley, 2017).

In terms of practical applications, there is a need for immobilization methods that reuse carriers after enzyme inactivation (Fraas and Franzreb, 2017). His-tagged enzymes combined with metal chelating supports offer the possibility to integrate enzyme purification and immobilization in a single step (Mateo et al., 2006). her advantage is the possibility to reuse the immobilization carrier after the enzyme inactivation (Fraas and Franzreb, 2017). In the present study, immobilized RrADH was desorbed and washed from the column with imidazole solution, replaced with fresh enzyme, and repeatedly used in subsequent acetophenone bioreduction batches.

## Conclusion

In this study, an effective dual biotransformation system by combining recombinant alcohol dehydrogenase and formate dehydrogenase was created and used for aldehyde and ketone bio-reduction. The main strategy was based on the use of poly-His tagged enzymes that can be reversibly immobilized on poly-histidine on metal-chelated carriers. Separate enzyme immobilization instead of co-immobilization on the same support enables the regeneration of an individual immobilized enzyme by replacement after inactivation. The protocol designed and tested herein serves as a model for the development of alcohol dehydrogenase-based biocatalytic systems with NADH regeneration.

## Data Availability Statement

The raw data supporting the conclusions of this article will be made available by the authors, without undue reservation.

## Author Contributions

SS, VŠ, JT, PF, VS, and MR: conceived and designed the experiments. VV, LM, ZL, ES, JB, and RK: performed the experiments. SS and VŠ: performed the data analyses. All authors contributed to the article and approved the submitted version.

## Conflict of Interest

The authors declare that the research was conducted in the absence of any commercial or financial relationships that could be construed as a potential conflict of interest.
